# Impact of biologics on growth in children with juvenile idiopathic arthritis

**DOI:** 10.1186/1546-0096-9-S1-P139

**Published:** 2011-09-14

**Authors:** F Uettwiller, G Pinto, M Polak, P Quartier

**Affiliations:** 1 Unité de Spécialités Pédiatriques, CHU Clocheville, Tours, France; 2 Endocrinologie Pédiatrique, Necker Enfants Malades, Paris, France; 3 Unité d'Immunologie, d'Hématologie et de Rhumatologie Pédiatriques, Necker Enfants Malades, Paris, France

## Background

Juvenile idiopathic arthritis (JIA) is sometimes associated with growth impairment due to inflammation and/or steroid treatment. Biologics allow a better control of inflammation and may therefore have a positive effect on growth.

## Aim

To assess growth of JIA children on biologics.

## Methods

JIA patients followed in one reference center, who were prebupescent at onset of biologic treatment, were included. We collected data about their growth and their disease (course, treatments, activity). Heights were expressed in standard deviations (SD) corrected with target height, at disease onset, at onset of biologics and at last follow-up.

## Results

100 patients were included. 29% were systemic. Mean age and height were 4.26 years (0.7; 13.7) and 0.31 SD (-2.47; 5.46) respectively at disease onset, 7.1 years (1.6; 15.7) and -0.24 SD (-3.63;2.90) at onset of biologics (p<0.05), 11 years (2.3;19.5) and -0.15 SD (-4.95;3.52) at last follow-up. 93 had received anti-TNFα, 35 anti-IL1, 12 anti-IL-6 and 9 CTLA4-Ig. 45% had an active disease. More than one biological agent was statistically linked with poor growth. At last follow-up, 10 children had a growth delay and 6 were treated with growth hormone.

## Conclusion

Growth delay was observed before biologics were started. On biologic treatment, growth velocity normalized, however there was no correction of growth delay. The discrepancy with previous studies, which showed a growth restoration on anti-TNF, might be linked to a higher proportion of systemic and severe polyarticular JIA patients. In a subset of patients, growth hormone treatment has to be discussed even at the era of biologics.

